# Development of an Oral Subunit Vaccine to Protect Rainbow Trout Against Infectious Hematopoietic Necrosis Virus (IHNV)

**DOI:** 10.1007/s10126-026-10728-w

**Published:** 2026-11-12

**Authors:** Borja Ordóñez-Grande, Mauricio Rojas-Peña, Oluwabusayo Israel Okeleye, Ainhoa Gómez-Quintanilla, Marlid Garcia-Ordoñez, Patricia Aceituno, Hamza Hassine, Maria del Mar Ortega-Villaizan, Luis Perez, Nerea Roher

**Affiliations:** 1 Institute of Biotechnology and Biomedicine (IBB), Universitat Autònoma de Barcelona, Cerdanyola del Vallès, 08193 Spain; 2 Department of Cell Biology, Physiology and Immunology, Universitat Autònoma de Barcelona, Cerdanyola del Vallès, 08193 Spain; 3 The Roslin Institute and Royal (Dick) School of Veterinary Studies, University of Edinbrugh, Edingrugh, UK; 4 Instituto de Investigación Desarrollo e Innovación en Bioteconología Sanitaria de Elche (IDiBE), Universidad Miguel Hernández, Alicante, 03202 Spain; 5 Centro de Investigación Biomédica en red de Bioingeniería, Biomateriales y Nanomedicina (CIBER-BBN), Cerdanyola del Vallès, 08193 Spain

**Keywords:** IHNV, Oral vaccination, Nanoparticle, Subunit vaccine, IFNγ

## Abstract

Infectious hematopoietic necrosis virus (IHNV) is a highly virulent pathogen of salmonids that causes severe mortality in aquaculture, particularly during early developmental stages. Current vaccination strategies rely mainly on injectable DNA vaccines, which are labour-intensive and unsuitable for large-scale immunization of juvenile fish. In this study, we developed and evaluated two recombinant nanostructured subunit vaccines based on the IHNV glycoprotein: IHNV^NP^ and IHNV-IFN^NP^, the latter incorporating interferon-gamma (IFNγ) as an immunomodulatory adjuvant. Both vaccines were produced in *Escherichia coli* as nanoparticles. In vitro assays using the rainbow trout macrophage-like cell line RTS11 showed efficient cellular uptake of both subunit vaccines and colocalization of IHNV-IFN^NP^ within acidic lysosomes, where antigen processing takes place. Gene expression analysis in RTS11 revealed that the IFNγ-adjuvanted vaccine significantly upregulated antiviral and immune-related genes associated with innate and adaptive responses, including *vig1*, *ip10*, *mx*, *ccl4*, *il-1β*, *mhcI*, and *mhcII*, compared with the non-adjuvanted formulation. Furthermore, an in vivo trial demonstrated that oral administration by intubation of IHNV-IFN^NP^ induced partial protective responses against IHNV challenge in rainbow trout fingerlings. In addition, ELISA and hindgut gene expression analysis in juvenile rainbow trout following oral intubation with IHNV-IFN^NP^ provided further evidence of the vaccine’s potential to induce mucosal immune responses. These findings indicate that an IFNγ-functionalized nanostructured subunit vaccine enhances immune activity against IHNV and represent a promising strategy for the development of practical, modular antiviral oral vaccines in aquaculture.

**Supplementary Information:**

The online version contains supplementary material available at https://doi.org/10.1007/s10126-026-10728-w.

## Introduction

Infectious hematopoietic necrosis virus (IHNV) is one of the most virulent viruses classified as notifiable on the watchlist of the World Organization for Animal Health (WOAH, 2024), which may cause 70–80% of mortality depending on the life-stage of the fish host (Kurath et al. 2013; Dixon et al. 2016). This disease affects salmonids, mainly at fry stage in spring and autumn (Moldal et al. 2025). IHNV is within the *Novirhabdovirus* genus, *Rhabdoviridae* family and has a single-stranded, negative-sense RNA genome (Morzunov et al. 1995). The viral glycoprotein (G-protein) mediates the viral entry into the host cells and is the main activator of the host innate immune system (Verjan et al. 2008). Therefore, it has become the major host-protective antigen of the virus to induce neutralizing antibodies (Ristow and Arnzen de Avila 1991).

One of the major bottlenecks to sustainable farmed fish are disease outbreaks (Reverter et al. 2020; FAO 2024). As production becomes more intensive, infectious diseases have been reported to become more deadly (Robinson et al. 2023). Thus, the lack of commercial therapeutics makes infections from virulent viruses the most devastating pathogen associated disease outbreaks (Kibenge 2019; Mugimba et al. 2021; Valero and Cuesta 2023). The prevalence of deadly infections is associated with an increased adaptation of the pathogen to their target host and prevailing environmental conditions (Valero and Cuesta 2023). An example is the increase in IHNV virulence in salmonids due to the pathogen’s genetic adaptations to the specific hosts (Balakhnina and Melnikov 2024).

Vaccines have emerged as an effective, environmentally friendly treatment to manage viral infections in the aquaculture industry (Ma et al. 2019), which boosts host’s immune system through the activation of recognition and elimination of invading pathogens (Aida et al. 2021), while reducing antibiotic usage in the aquaculture sector. However, commercial vaccines are mainly directed against bacterial pathogens and least against viral pathogens (Ma et al. 2019; Mondal and Thomas 2022). The most popular methods of vaccine administration are injection and immersion, which are known to cause stress with negative consequences on fish welfare (Bøgwald and Dalmo 2021; Kim et al. 2023). Contrary, oral vaccines are considered a better alternative, more desirable administration method which avoids labour-intensive, time-consuming and stress caused by injection procedures (Gorgoglione et al. 2023). In addition, the presence of professional peripheral antigen-sensing cells in the mucosal tissues allows orally administered antigens to stimulate immune system response (Liu et al. 2024). However, gastrointestinal pH barrier, protective dose optimisation, oral tolerance chance and proper stimulation of antigen presenting cells (APCs) are some of the drawbacks oral vaccines must face (Embregts and Forlenza 2016; Adams 2019; Ma et al. 2019; Coffey et al. 2021). In the case of IHN, an injectable commercial DNA vaccine (Apex-IHN) is available and only marketed in Canada (Alonso and Leong 2013; Rojas-Peña et al. 2026), however, vaccination against IHNV is needed within the first two months of salmonids life, therefore an orally administered vaccine urges to be developed (Dixon et al. 2016; Yong et al. 2019; Gorgoglione et al. 2023). From a research perspective, oral vaccine development has primarily focus on recombinant and DNA vaccines-based approaches (Rojas-Peña et al. 2026). In the case of IHNV, most of the studies have focused on DNA vaccines with good results (Alonso et al. 2011; Xu et al. 2017; Jones et al. 2020; Marsella et al. 2022; Wu et al. 2023; Zhao et al. 2024; Doumayrou et al. 2025), followed by a small number investigating recombinant subunit vaccines (Engelking and Leong 1989; Verjan et al. 2008; Hua et al. 2020; Gorgoglione et al. 2023; Luo et al. 2026). More recently, mRNA vaccines have gained attention as a novel approach (Wang et al. 2025).

Subunit vaccines provide the possibility to safely produce heterologous expressed antigens against pathogens that are difficult to culture, with a broad applicability that protects against individual or multiple pathogens by incorporating different pathogens antigens. In addition, subunit vaccines are highly characterized, can incorporate non-natural components, can allow for non-refrigerated transport and storage, while also pose no risk of disease or replicability in the host (Du et al. 2022). Unfortunately, one of the major drawbacks on subunit vaccines is the weaker immune response compared to other vaccines, like inactivated or live attenuated pathogens, usually related to the low number of immunogenic components associated to the vaccine. Thus, to promote long-lasting protection in vaccinated fish, these vaccines need multiple or booster doses in addition to be combined with adjuvants (Hansson et al. 2010; Dalmo et al. 2016). An adjuvant is a substance or formulation that promotes vaccine efficacy by enhancing host response to vaccine antigens through essential immune signal activation (Rojas-Peña et al. 2026). Immunoregulatory proteins, such as cytokines, become a natural target as vaccine adjuvant as their essential role is to mediate in cellular communication within the immune system. One cytokine that generates a potent antiviral response is interferon (IFN), which adjuvant properties have been studied in vitro in different fish cell lines (Martin et al. 2007; Kuo et al. 2016; Bedekar et al. 2018; Morales-Lange et al. 2021). IFNs can activate innate immune defence response against viral infections (type I) and enhance cell-mediated immunity (type II). While IFNγ (type II) can promote antigen presentation by up-regulating expression of both MHC class I and MHC class II molecules, type I IFNs promotes mainly MHC class I (Robertsen 2006; Casani et al. 2009; Toporovski et al. 2010; Zou and Secombes 2011). IFNγ activates macrophages and T lymphocytes, mediates cell proliferation, apoptosis, inflammation and leukocyte trafficking. It has been coupled with antigen protein in nanostructured recombinant vaccines against spring viraemia carp virus (SVCV), developing a strong immune response in vitro and protecting zebrafish against SVC virus with a RPS of 76% after intraperitoneal (i.p.) injection (Rojas-Peña et al. 2022). In the same way, Aceituno et al. (2025) developed a vaccine against viral haemorrhagic septicaemia virus (VHSV) by coupling IFNγ with VHS glycoprotein that activated local and systemic antiviral response after oral intubation and in-feed oral administration, producing neutralizing antibodies. Both vaccines were produced in the form of Inclusion Bodies (IBs), which are *de novo* insoluble protein aggregates formed after the forced expression of heterologous genes in bacterial factories. Usually seen as a drawback in high-yield soluble protein production, IBs have been described as functional materials with high potential in industry and biomedicine applications, by naturally penetrating mammalian cells, targeting specific receptors and working as secretory granules (Rinas et al. 2017). In addition, IBs have shown resistance to different pH conditions (2 and 10) and high temperatures (100 °C) (Rojas-Peña et al. 2022; Aceituno et al. 2025), while reducing viral load regarding the controls (Aceituno et al. 2025). Compared to other subunit vaccines, IBs do not need encapsulation and can be in-feed extruded for oral delivery, reducing the cost of vaccination.

In the present study, we used a well-known vaccine platform against viral disease to develop two subunit vaccines, IHNV^NP^ and IHNV-IFN^NP^. We assessed its morphology, functionality and colocalization in in vitro assays using a rainbow trout macrophages-like cell culture (RTS11). Also, macrophage-like immune response to vaccine administration was measured in vitro. Furthermore, an in vivo assay was performed to assess vaccine protection after infection challenge when administered orally by intubation in rainbow trout fingerlings. Additionally, the potential of the modular IHNV vaccine to induce mucosal immune responses in juvenile trouts was investigated by analysing the expression of immune-related genes and the levels of IHNV-specific antibodies in the hindgut following oral administration.

## Materials and Methods

### Design, Production and Quantification of IHNV^NP^ and IHNV-IFN^NP^

#### Viral Strains and Plasmids

A modular vaccine was designed based on infectious hematopoietic necrosis virus glycoprotein (AA 270–460, NCBI acc.no: AOG30717.1). Literature research was performed to design the specific epitope region for IHNV antigen (Xu et al. 1991; Lapatra et al. 1995; Ammayappan et al. 2010; Wang et al. 2016; Guo et al. 2018; Zhao et al. 2019). Specifically, this region includes three already described antigenic regions (Kim et al. 2007). IHNV-IFN^NP^ vaccine included an *Oncorhynchus mykiss* interferon gamma (IFNγ) module (AA 25–180, Uniprot acc. no: Q5QSL2) bound by a linker peptide GGSSRSS (Argos 1990). GeneArt service (Thermo Scientific) was used to produce the pET22b constructs with *Escherichia coli* (*E. coli*) codon optimization and a C terminal polyHis tag. Red fluorescent protein (iRFP^NP^) was used as non-immune relevant control. Heat shock transformed *E. coli* BL21/DE3 (Novagen) was used for recombinant protein expression of both vaccines (IHNV^NP^ and IHNV-IFN^NP^) and non-immune relevant control protein (iRFP^NP^).

#### Production and Purification

**Fig. 1:**
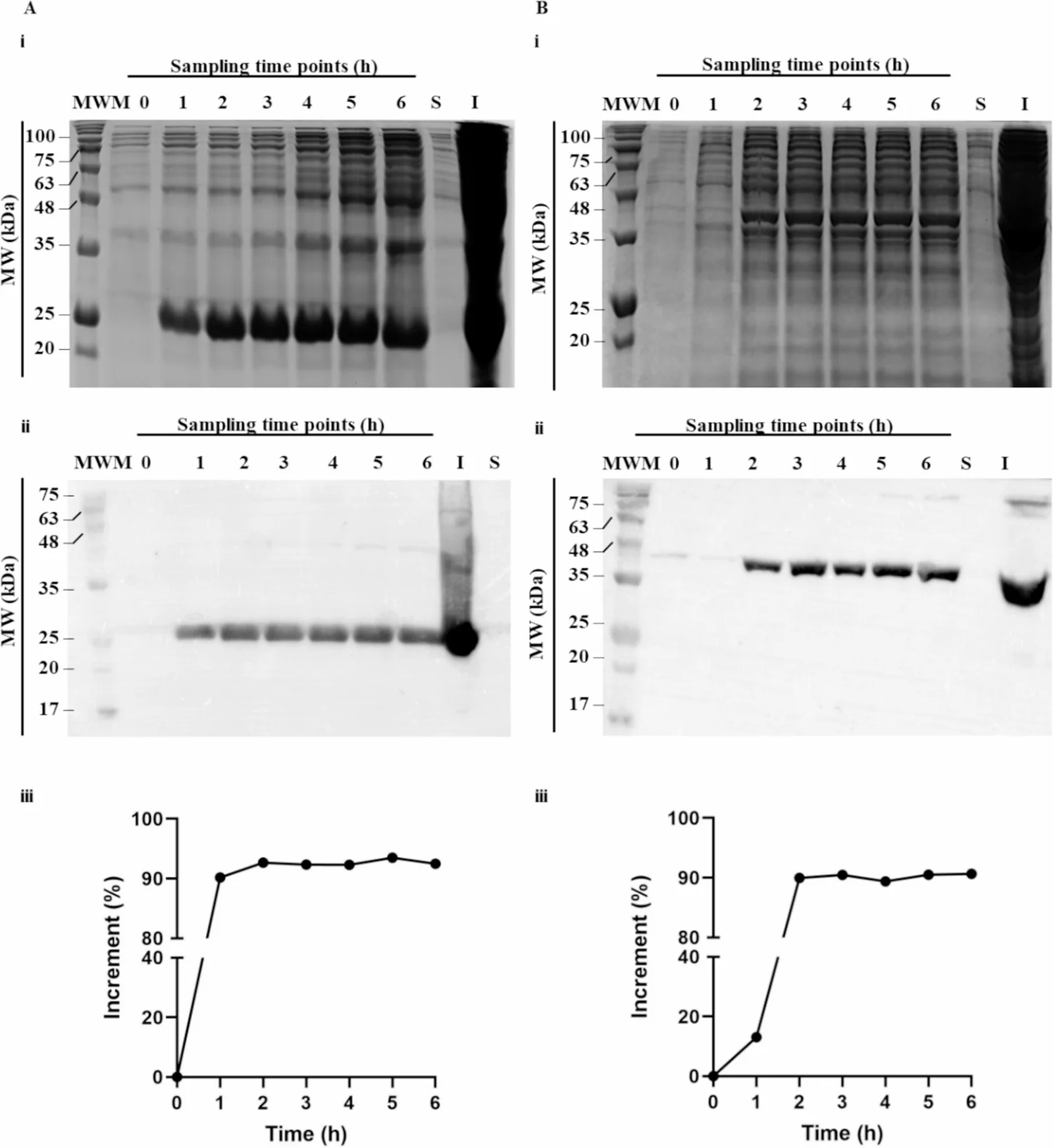
Induction test of IHNV^NP^ (**A**) and IHNV-IFN^NP^ (**B**). SDS-PAGE Coomassie Blue (i) and Western Blot (ii) with molecular weight marker (MWM, 17–100 kDa), Sampling times (0, 1, 2, 3, 4, 5 and 6 h), Soluble fraction (S) and Insoluble fraction (I). Percentage of increment (iii) of production in the different sampling times in respect to time zero

IHNV^NP^, IHNV-IFN^NP^ and iRFP^NP^ were produced and purified in the form of inclusion bodies (IBs) according to the method described by Thwaite et al. (2018) with Rojas-Peña et al. (2022) modifications. Briefly, *E. coli* was grown overnight (O/N) in LB medium (Gibco) with 100 µg/mL ampicillin (Sigma-Aldrich) and induced protein expression with 1 mM isopropyl β-D-1-thiogalactopyranoside (IPTG, Panreac) for 3 h at 37 °C in 250 rpm. Enzymatic and mechanical disruption was used for purification, according to Torrealba et al. (2016) methodology. Briefly, cell culture was treated with 1.5 µg/mL lysozyme (Roche), protease inhibitor cOmplete EDTA-free tablets, and 0.4 mM phenyl-methanesulfonyl fluoride (PMSF, Roche) for 2 h at 37 °C in 250 rpm, then O/N frozen at -80 °C. After thawing, suspension was treated with 0.2% (v/v) Triton X-100 for 1 h at RT and 125 rpm followed by a 1 µg/mL DNAse I (Roche) and 1 mM MgSO_4_ (Sigma-Aldrich) treatment for 1 h at 37 °C and 125 rpm. Nanoparticles (NPs) were harvested by centrifugation at 15,000 x*g* for 15 min at 4 °C and resuspended in a 1/10 original volume of 50 mM Tris HCl pH 8, 100 mM NaCl, 1 mM EDTA buffer. Lastly, remaining viable bacteria was eliminated by freeze-thaw cycles. LPS was not directly quantified nor removed from vaccine productions. Production time was determined by an induction test. Following the same production process above described, a control sample was taken before induction with IPTG, and following samples were retrieved at 1, 2, 3, 4,5 and 6 h post-induction. A final sampling was done O/N for separate soluble and insoluble fractions. All samples were sonicated for 20 s.  (1 s ON/0.5 s OFF) at 20% amplitude (~ 200kV) for 3 cycles. After centrifugation at 10,000 x*g* at 4 °C for 5 min, supernatant was discarded, except for O/N sample, and pellet was resuspended in loading buffer for quantification (Fig. 1).

#### Quantification and Fluorescent Labelling

IHNV^NP^, IHNV-IFN^NP^ and iRFP^NP^ were quantified by SDS-PAGE Coomassie Brilliant Blue R250 staining (Sigma-Aldrich). Different amounts of bovine serum albumin (Sigma-Aldrich) were used to make a standard curve. After staining, polyacrylamide gels were scanned using Chemidoc Imagin System (Bio-Rad) and the final concentration was calculated using Image Lab 6.0.1 (Bio-Rad). Molecular weight was confirmed by Western Blot using an anti-His-tag antibody (Genscript A00186-100). Vaccine NPs were covalently conjugated with Atto-488 NHS ester (Sigma-Aldrich) following manufacturer’s instructions. Fluorescent IHNV^NP^ and IHNV-IFN^NP^ were used for flow cytometry. Nanodrop ND-100 (Thermo Scientific) was used to calculate labelling efficiency.

### Morphometric Characterization of IHNV^NP^ and IHNV-IFN^NP^

NPs morphology was determined using Field Emission Scanning Electron Microscopy (FESEM, Zeiss Merlin). A final concentration of 100 µg/mL suspension of IHNV^NP^ and IHNV-IFN^NP^ in sterile MQ H_2_O were deposited onto silicon chips and air-dried O/N. Size of at least 200 NPs was measured using ImageJ (National Institutes of Health) Fiji software package (Schindelin et al. 2012). Histograms of size normal distribution were plotted with Past 4.04 software (Hammer et al. 2001).

### In Vitro Functional Characterization of IHNV^NP^ and IHNV-IFN^NP^ Subunit Vaccines

#### Cell Culture

Rainbow trout (*O. mykiss*) spleen monocyte/macrophage-like cell line (RTS-11) (Ganassin and Bols 1998) was cultured in Leibovitz’s 15 medium + GlutaMAX™ (Gibco), 20% FBS (Gibco), 1% (v/v) antibiotic/antimycotic (Gibco) at 21 °C without CO_2_.

#### IHNV^NP^ and IHNV-IFN^NP^ Uptake

Fluorescent NPs above described (2.1.3) were used to address vaccine cellular internalization. RTS11 cells at a 70–80% confluence were incubated in minimal medium (0% FBS) for 2 h before being exposed to different doses and times. For dose response assays, IHNV^NP^ and IHNV-IFN^NP^ were incorporated at 40, 80 and 160 µg/mL and incubated for 14 h. For time-course assays, IHNV^NP^ and IHN-IFN^NP^ were added at 80 and 160 µg/mL and incubated for 12 and 24 h. After NPs treatment, Tryple Express (Gibco) was used to detach cells. Cell suspension was centrifuged at 300 x*g* for 5 min at RT and cell pellets were resuspended in PBS 1x (Sigma-Aldrich) to perform flow cytometry (CytoFLEX LX, Beckman Coulter). BD Via-Probe™ (Becton Dickinson) was used as non-viable cell marker. At least 10,000 events were recorded, analysed using CytExpert 2.4 (Beckman Coulter) and the results plotted with Prism 8.01 (GraphPad Prism). The gating strategy is shown in Sup Fig. 4.

#### IHNV-IFN^NP^ Cellular Processing

To address cellular processing of vaccine NPs, labelled IHNV-IFN^NP^ was up taken by RTS11 cells at 160 µg/mL in poly-D-lysine-treated 35 mm µ-Dish (Ibidi GbmH), using the same procedures as described in 2.3.2. After 24 h, cells were treated with LysoTracker Red DND-99 (Thermo Fisher Scientific) to stain lysosomes in red. Nuclei stain was done using Hoechst 33​342 (Thermo Fisher Scientific). Images were taken at Universitat Autònoma de Barcelona microscopy service (SMiDRX) using a confocal microscope (Confocal Zeiss LSM980, Carl Zeiss AG, Germany) and were processed with ImarisViewer v10.0.1 and Imaris software v7.2.1 (Oxford Instruments).

#### Gene Expression

RTS11 as immune cell line were exposed to NPs to address gene expression of immune response related genes. Cells were cultured and exposed to NPs as above described (2.3.1 and 2.3.2). Four independent biological replicates, derived from different passages, were used to ensure the robustness of statistical analysis. For gene expression, cells were incubated with controls and NPs for 24 h as an initial screening time point to assess whether NPs were able to induce an antiviral response. RTS11 was incubated with IHNV^NP^ and IHNV-IFN^NP^ at 160 µg/mL. iRFP^NP^ at 20 µg/mL was used as protein production control, LPS from *E. coli* O111:B4 strain (Sigma-Aldrich) at 10 µg/mL as pro-inflammatory response control, Poly(I:​C) (Sigma-Aldrich) at 10 µg/mL, as anti-viral response control and a control without stimulus (PBS 1x). Primer sequences were previously designed and tested by Thwaite et al. (2018) and Aceituno et al. (2025) (Supplementary Table 1). For reference genes, Elongation factor 1 alpha (*ef1-α*) and Beta actin (*β-actin*) were tested, while 9 genes were selected to address immune response to viral infection (*vig1*,* mx*,* ccl4*,* il1β*,* ip10*,* ifngr2*,* mhcI*,* mhcII* and *igm*), and 10 genes were selected to address gut immune response 30 days after oral intubation (*mx*,* il10*,* cd4*,* cd8*,* mhcI*,* mhcII*,* foxp3*,* tgfβ*,* igm* and *igt*).

### In Vivo Functional Characterization of IHNV-IFN^NP^ Subunit Vaccine

#### Fish Husbandry

Two in vivo trials in rainbow trout (*O. mykiss*) were conducted one at Universitat Autònoma de Barcelona (UAB) and another at Universidad Miguel Hernandez (Spain) facilities to determine IHNV-IFN^NP^ vaccine functionality. At UAB wet lab facilities trouts of 52.3 ± 5.5 g were acclimated for 10 days and orally intubated to assess mucosal immunity and systemic protection after 30 days of oral immunization. Fish were placed in tanks at 16 ± 1 °C under a recirculating water system, 12/12 light/dark photoperiod and fed twice a day for a total of 2% body weight with a commercial diet. At UHM, a small-scale oral intubation trial was performed to assess correlates of protection after 30 days post-infection (dpi). Trouts with an average weight of 3–3.5 g were maintained in aquariums in a recirculating water system at 13 ± 1 °C, 12:12 light/dark photoperiod and fed twice a day for a total 2% of their body weight with a commercial diet. Fish were acclimated to the facilities for at least 2 weeks prior to the experiment. All animal proceedings were carried out according to the International Guiding Principles for Research Involving Animals (EU 2010/63), Royal Decree RD 53/2013 and the Research Ethics Committee of the UAB, UHM and the Animal Welfare Body.

#### Oral Intubation, Mucosal Immunity and IHNV Infection Challenge

A preliminary assessment of mucosal immunity and a viral challenge involving 10 fish per experimental condition were conducted to evaluate vaccine-induced protection. IHNV-IFN^NP^, iRFP^NP^ and Control were administered by intubation as described by Ji et al. (2018) with some modifications. Fish were anesthetized with MS-222 (50 mg/L, Sigma-Aldrich). Rainbow trout were intubated with a volume of 100 µL using a 25G syringe with a thin silicon tube placed over the needle and introduced in the mouth until reaching the oesophagus. Once intubated, fish were left to recover in aerated containers before being returned to the aquarium. During this recovery period, fish were under observation to detect any regurgitation. Intubation procedure was performed within three consecutive days to deliver a cumulative dose of 125 mg/kg of IHNV-IFN^NP^ or iRFP^NP^. During dose preparation, NPs were resuspended in PBS 1x. To help reduce protein aggregation a thorough mixing was performed prior oral gavage. Control fish were intubated with PBS 1x. Hindguts were collected 30 days after oral intubations and placed in RNAlater (Invitrogen). Samples were taken from control groups (PBS and iRFP^NP^; *n* = 7) and the IHNV modular vaccine group (*n* = 10). For the IHNV challenge trial, at 30 days post immunization, trout fingerlings were anesthetized with MS-222 (50 mg/L, Sigma-Aldrich) and intraperitoneally (i.p.) injected with 50 µL of Infectious hematopoietic necrosis virus (IHNV) at a 1·10^6^ titer of Plaque-Forming Unit (PFU) per fish. Viral dose was determined based on previous challenge test with the IHN virus stock using a small number (*n* = 5) of equivalent size fish. Trout mortality rate was recorded daily for the next 20 days. Temperature was kept at 13 °C over the course of the challenge.

### RNA Extraction, cDNA Synthesis and RT-qPCR

Hindgut tissues were homogenized using a Polytron PT E 1600 (KINEMATICA AG) and total RNA was extracted using TriReagent (Sigma-Aldrich) according to manufacturer’s instructions and quantified using nanodrop ND-1000 (Thermo Fisher Scientific). cDNA was synthesized from 150 ng (PBLs) or 750 ng (tissue or cell culture) high-quality total RNA using iScript cDNA synthesis kit (Bio Rad). Quantitative real-time PCR (RT-qPCR) was performed at 60 °C in a CFX384 real-time PCR detection system (Bio Rad) using iTaq Universal SYBR Green Supermix kit (Bio Rad) following the manufacturer’s instructions. Each PCR mixture consisted of 5 µL SYBR Green Supermix, 0.4 µM specific primers, 2 µL diluted cDNA (1:10) and 2.6 µL water (Sigma-Aldrich) in a final volume of 10 µL. All tested genes were diluted at a 1:10 factor. Elongation factor 1 alpha (*ef1-α*) was selected as reference gene in comparison to Beta-actin (*β-actin*). Primer sequences are listed in Supplementary Table 1. Data was analysed using the Livak method (Livak and Schmittgen 2001).

### Serum Specific Antibody Titer

The ability of IHNV-IFN^NP^ to stimulate the adaptive immune system was determined by indirect ELISAs. Specific anti-IHNV IgM titer was detected in serum of orally intubated trouts with 125 mg NPs/Kg fish in 50 g fish after 30 days post-vaccination (n = 10) and in 3 g fish before infection challenge (n = 4). The antibody titer was assessed as previously described (Puente-Marin et al. 2019). Briefly, a positive serum control from IHNV-challenged survivors was incorporated and four serum dilutions (1/30, 1/60, 1/120, 1/240) were tested. The primary antibody used was an anti-trout IgM hybridoma clone 1.14 (Deluca et al. 1983) and a monoclonal rabbit anti-mouse conjugated with peroxidase (anti-rabbit IgG, γ-chain specific) (A1949, Sigma-Aldrich) was used as secondary antibody. Detection was performed using 3, 3.5’,5’ tetramethylbenzine (TMB) substrate reagent set (BD Biosciences), and the absorbance was measured at 450 nm on a microplate reader (VICTOR3, PerkinElmer).

### Statistics

GraphPad Prism 8.01 (GraphPad) was used for statistics analysis. Group mean comparison *versus* control was analysed by One-way ANOVA test, followed by Dunnett’s multiple comparison test, or unpaired one-sided t-test with Welch’s correction for unequal variances, or unpair nonparametric Mann-Whitney test. Data are shown as mean ± standard deviation (SD) and ns: non-significant; *p < 0.1; **p < 0.05; ***p < 0.01; ****p < 0.001 was considered statistically significant.

## Results

### Production Yields and Morphological Characterization of IHNV^NP^ and IHNV-IFN^NP^

Two recombinant nanostructured modular subunit vaccines were produced and purified in the form of IBs using non-chromatographic methods. Induction test allowed to check for protein production and to determine optimal production time (Fig. 1). Flask production yields were 206.56 mg/L for IHNV^NP^ and 296.32 mg/L for IHNV-IFN^NP^.

**Fig. 2:**
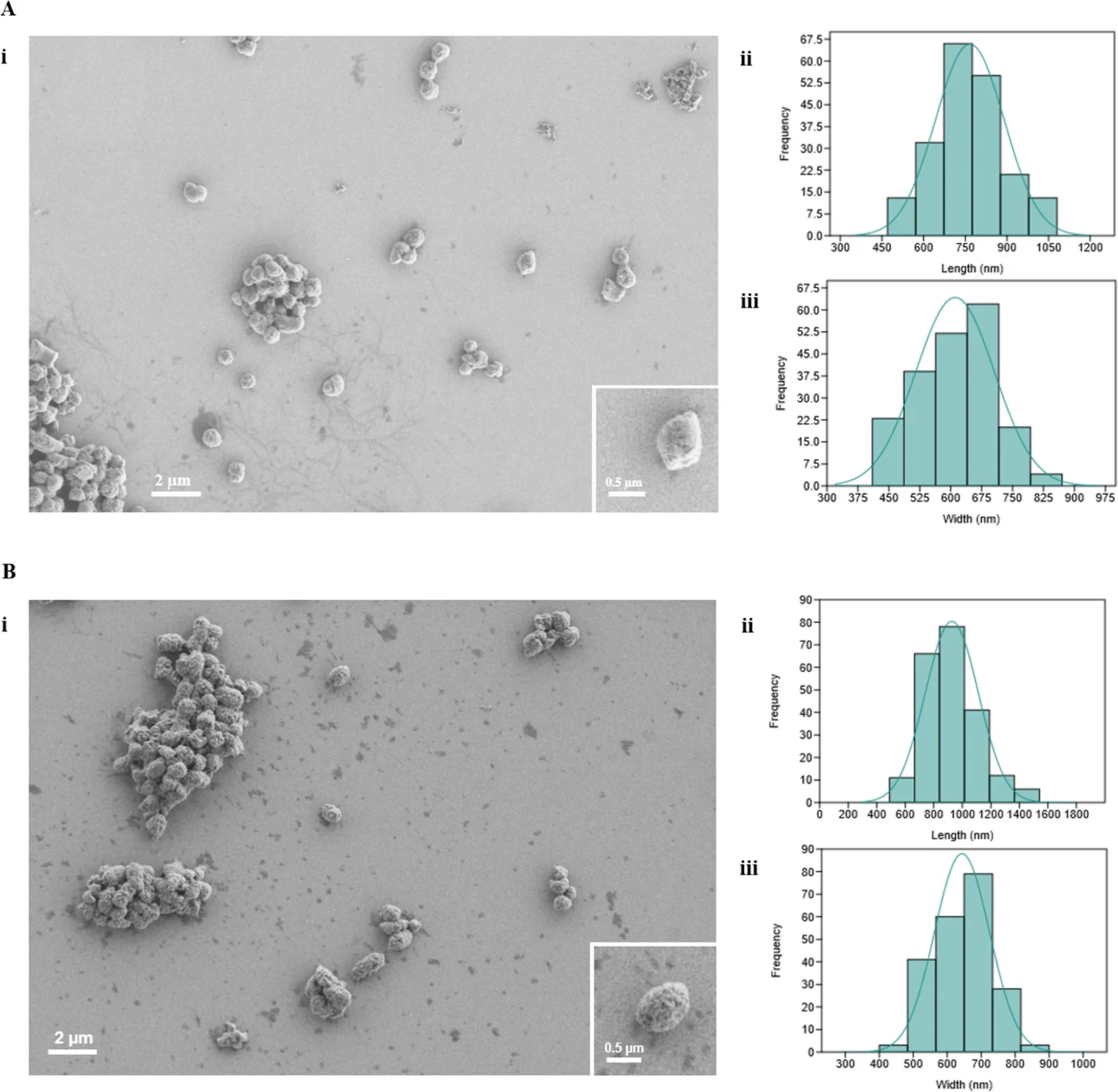
Characterization of IHNV^NP^ (**A**) and IHNV-IFN^NP^ (**B**) under Field Emission Scanning Electron Microscopy (FESEM). Size distribution histograms (A, *n* = 200; B *n* = 210) of length (ii) and width (iii) measurements of nanostructured proteins

NPs morphology and size comparison were assessed using FESEM (Fig. 2). After measuring a representative sample of at least 200 particles per production, IHNV^NP^ average length was 766.15 ± 123.23 nm and average width was 610.60 ± 95.22 nm. For IHNV-IFN^NP^ average length was 926.31 ± 185.83 nm and average width was 643.46 ± 80.90 nm. Both NPs showed a porous surface with an ellipsoid shape with a mix of small and rounded particles clustered together. IHNV-IFN^NP^ and IHNV^NP^ nanoparticles molecular weight was 41.34 ± 1.55 kDa vs. 29.83 ± 0.63 kDa, respectively (Fig. 2).

### Subunit Vaccine Uptake by RTS11

**Fig. 3:**
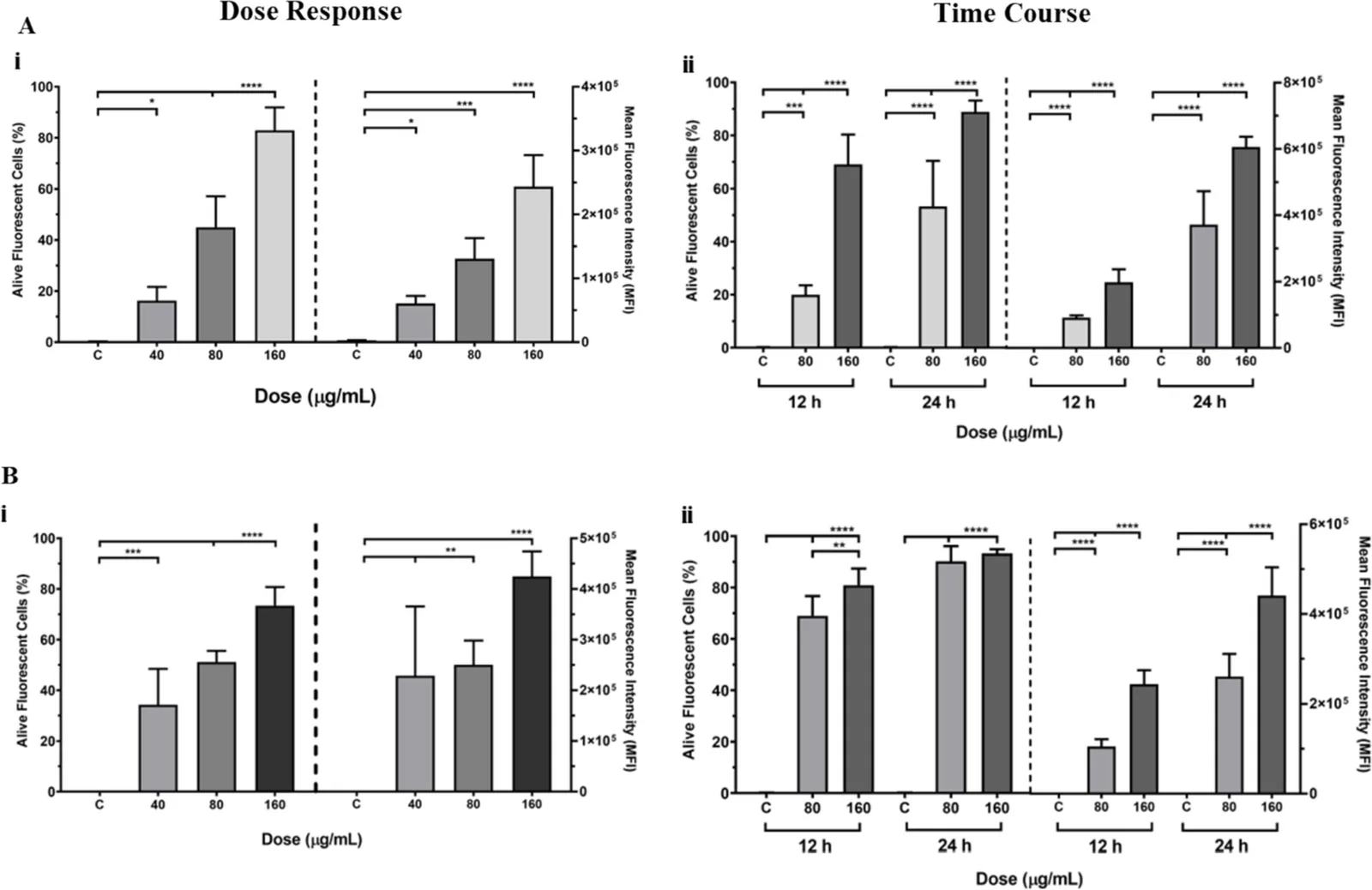
Uptake of IHNV^NP^ (**A**) and IHNV-IFN^NP^ (**B**) by RTS11 cells evaluated by flow cytometry. Dose response (i) for 40, 80 and 100 µg/mL of fluorescently labelled nanostructured protein after 14 h. Time course (ii) for 80 and 160 µg/mL of fluorescently labelled nanostructured protein after 12 and 24 h. The left Y-axis represents the percentage of fluorescent-positive viable cells (%), right Y-axis represents the mean fluorescence intensity (MFI). Differences between means were analysed by a one-way ANOVA with Dunnett’s multiple comparisons test, treatments versus control. Significance levels *p < 0.05; **p < 0.01; ***p < 0.001; ****p < 0.0001

Labelled IHNV^NP^ and IHNV-IFN^NP^ internalization was measured by a dose-response assay in RTS11 cell culture (Fig. 3). In both cases, NPs uptake and Mean Fluorescence Intensity (MFI) increased with the dose. All doses (40, 80 and 160 µg/mL) showed a significant increase compared to the control. Both NPs were up taken by 50% of the cells at 80 µg/mL dose.

For time-course assays, labelled IHNV^NP^ and IHNV-IFN^NP^ at 80 and 160 µg/mL were up taken by RTS11 after 12 and 24 h (Fig. 3). Both NPs showed a significant increase regarding the control at both times. In addition, an internalization above 90% after 24 h was measured in both NPs at 160 µg/mL. In the case of IHNV-IFN^NP^ this mark was achieved also at 80 µg/mL dose at the same timing. MFI values were similar to the dose response assay for 12 h time point, and significantly higher at 24 h. In this sense, IHNV-IFN^NP^ showed similar Alive Fluorescent Cell percentage after 24 h, though MFI was significantly higher in 160 µg/mL than 80 µg/mL dose, indicating possible multiple uptakes per cell.

### IHNV-IFN^NP^ Uptake and Processing by RTS11

**Fig. 4:**
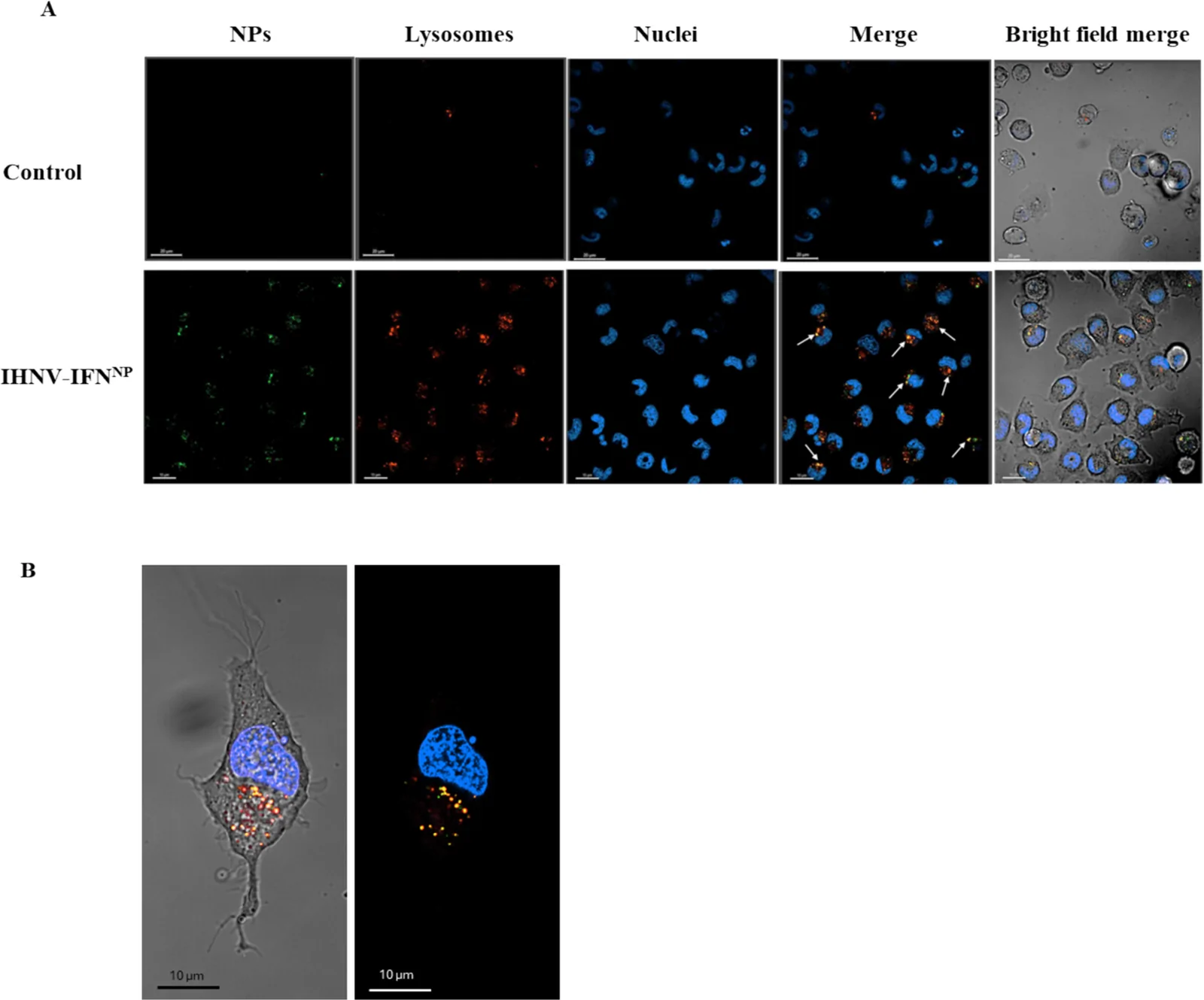
Colocalization of fluorescently labelled IHNV-IFN^NP^ and acidic lysosomes in RTS11 cells at 160 µg/mL after 24 h exposure under confocal microscopy. Cell culture view (**A**) and individual cell view (**B**) of RTS11 cell culture. Nuclei (blue), Lysosomes (red), IHNV-IFN^NP^ (green). White arrows indicate colocalization of nanostructured protein in lysosomes (yellow-orange)

NPs internalization and processing pathway were assessed in RTS11 with LysoTracker red DND-99 and Hoechst 33342 staining. After a 24 h, Atto-488 labelled IHNV-IFN^NP^ (Green) was colocalized in acidic lysosomes (Red) of macrophage-like cell line RTS11 (Fig. 4A). Colocalization areas are shown in bright yellow-orange colour in merge images (Fig. 4B).

### Gene Expression Analysis of RTS11 Exposed to NPs

**Fig. 5:**
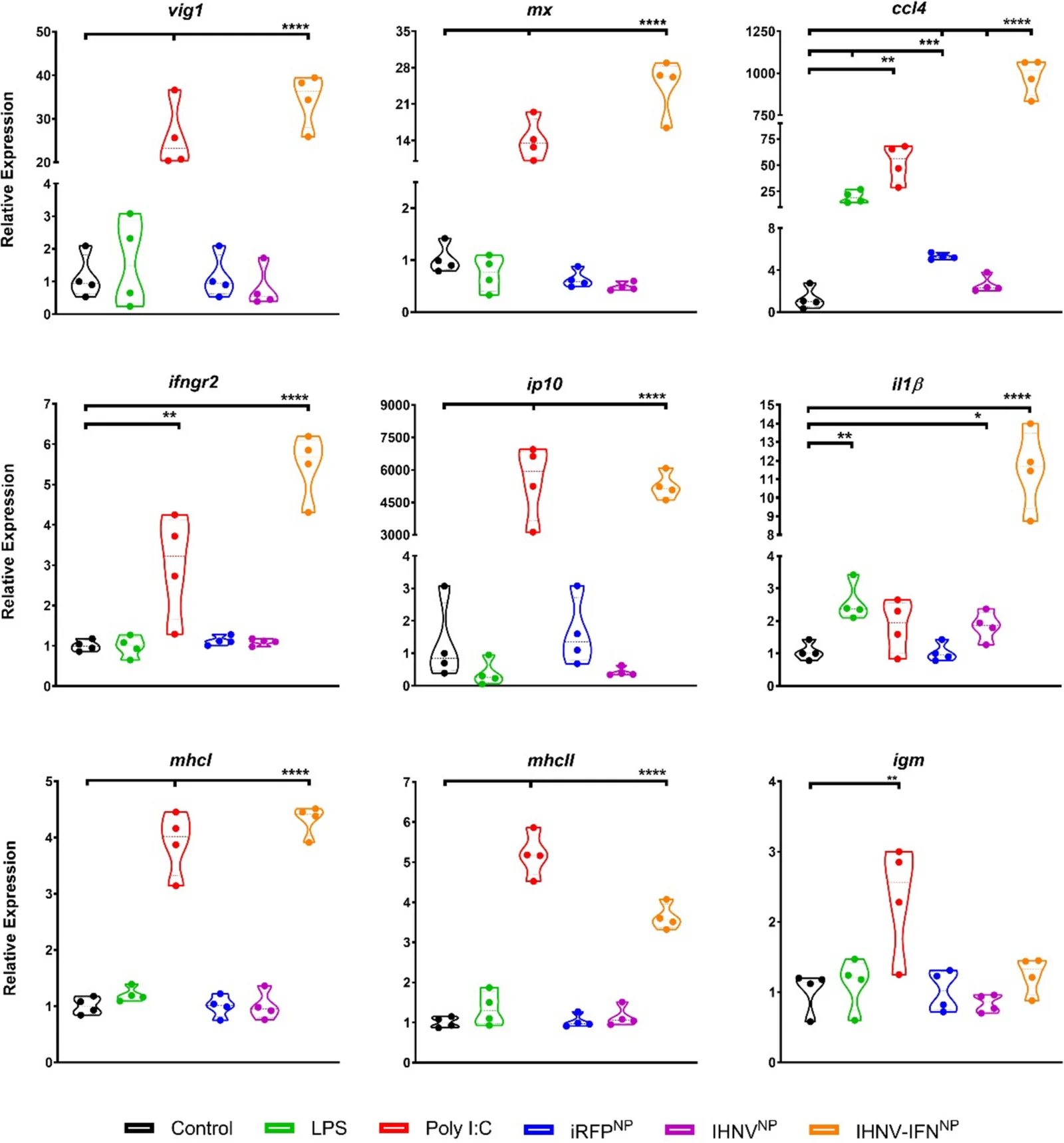
Gene expression of RTS11 cells treated with IHNV^NP^ and IHNV-IFN^NP^. RTS11 were 24 h exposed to: PBS 1x (black), 10 µg/mL LPS (green), 40 µg/mL Poly I:​C (red), 20 µg/mL iRFP^NP^ (blue), 160 µg/mL IHNV^NP^ (purple), 160 µg/mL IHNV-IFN^NP^ (orange). Data are mean ± SD (*n* = 4). Differences between means were analysed by a one-way ANOVA with Dunnett’s multiple comparisons test, treatments versus control, unpaired one-sided t-test with Welch’s correction for unequal variances or unpair nonparametric Mann-Whitney test. Significance levels *p < 0.05; **p < 0.01; ***p < 0.001; ****p < 0.0001

RTS11 cell culture was treated with IHNV^NP^ and IHNV-IFN^NP^ for 24 h at a 160 µg/mL dose each, considering the results obtained in dose response and time course assays (Fig. 5). In parallel, RTS11 were exposed to 20 µg/mL iRFP^NP^, 40 µg/mL Poly (I:​C), 10 µg/mL LPS and a control without treatment. IHNV-IFN^NP^ treatment showed the highest upregulation in *vig1*, *mx*, *ccl4*, *ifngr2* and *il1β* genes expression, Poly(I:​C) treatment showed the highest expression of *ip10* and *mhcII*, while both treatments showed similar expression in *mhcI*. IHNV^NP^, iRFP^NP^, LPS and Control treatments remained at lower relative expressions compared to IHNV-IFN^NP^ and Poly(I:C) treatments.

### Mucosal and Systemic Immunity

**Fig. 6:**
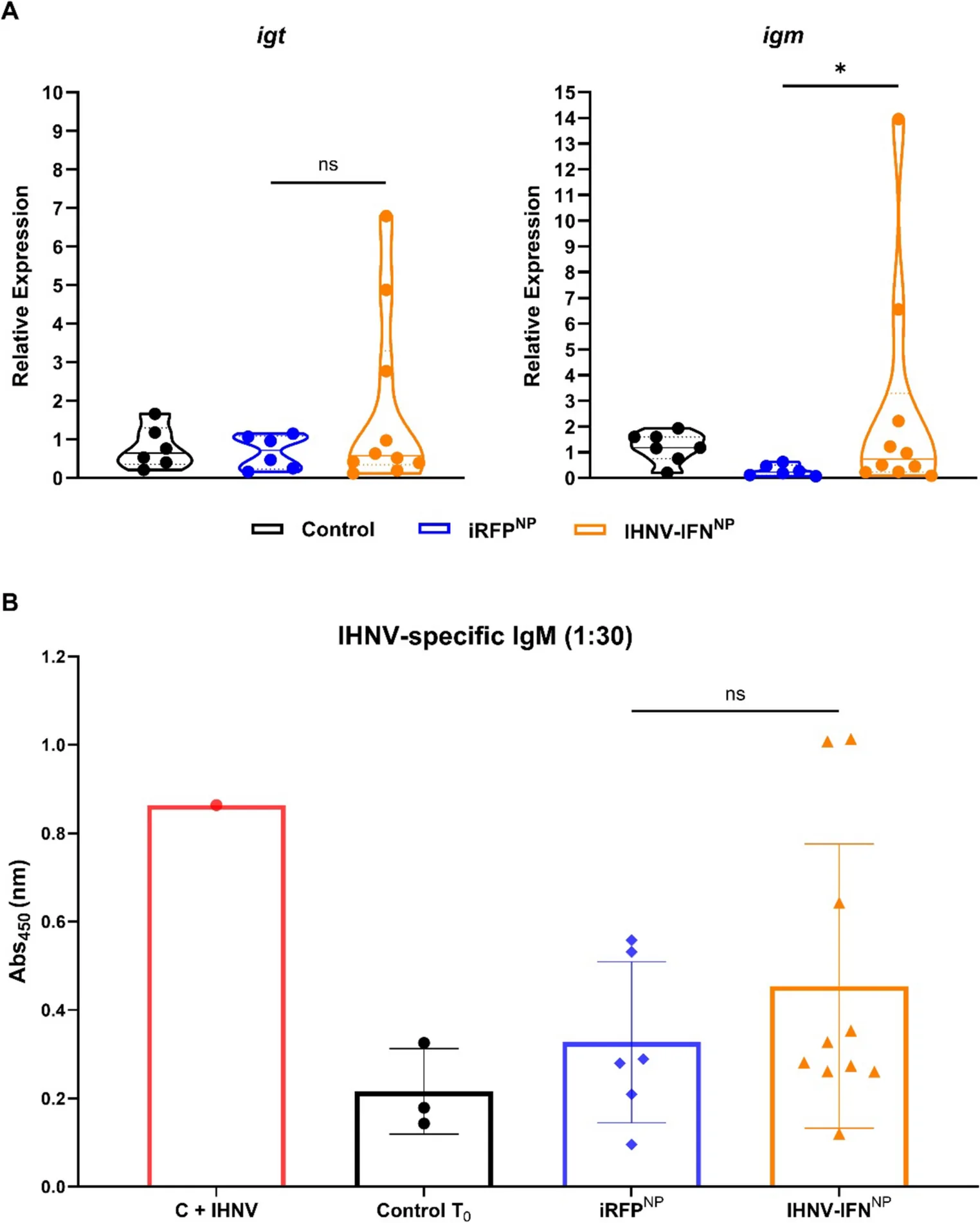
Mucosal and systemic immunity promotion after IHNV-IFN^NP^ oral intubation. (**A**) Gut gene expression after oral intubation and. (**B**) Presence of specific anti-IHNV IgM sera diluted 1:30 30 days post-vaccination with Control (black), iRFP^NP^ (blue), IHNV-IFN^NP^ (orange) and IHNV infection survival fish positive control (red). Data are mean ± SD (*n* = 10). Differences between means were analysed by a one-way ANOVA with Dunnett’s multiple comparisons test, treatments versus control, unpaired one-sided t-test with Welch’s correction for unequal variances or unpair nonparametric Mann-Whitney test. Significance levels *p < 0.05; **p < 0.01; ***p < 0.001; ****p < 0.0001

A preliminary trial to assess mucosal immunity and systemic immunity promotion was performed using oral intubation. We demonstrated that oral intubation promoted gut mucosal immunity by upregulating *igm* and *igt* expression in IHNV-IFN^NP^ vaccinated fish. While *igm* expression showed significant differences regarding the vaccine production control (iRFP^NP^), increased *igt* expression was observed in 30% of fish vaccinated with IHNV-IFN (3 out of 10), although no significant differences were detected between groups (Fig. 6A). Other mucosal immunity assessed genes are included at Supplementary Fig. 1. Similar to gut mucosal *igm* and *igt* expression, sera IgM titer slightly increased showing a tendency of systemic protection by IHNV-IFN^NP^ vaccine (Fig. 6B).

### IHNV Infection Challenge Survival Rates

**Fig. 7:**
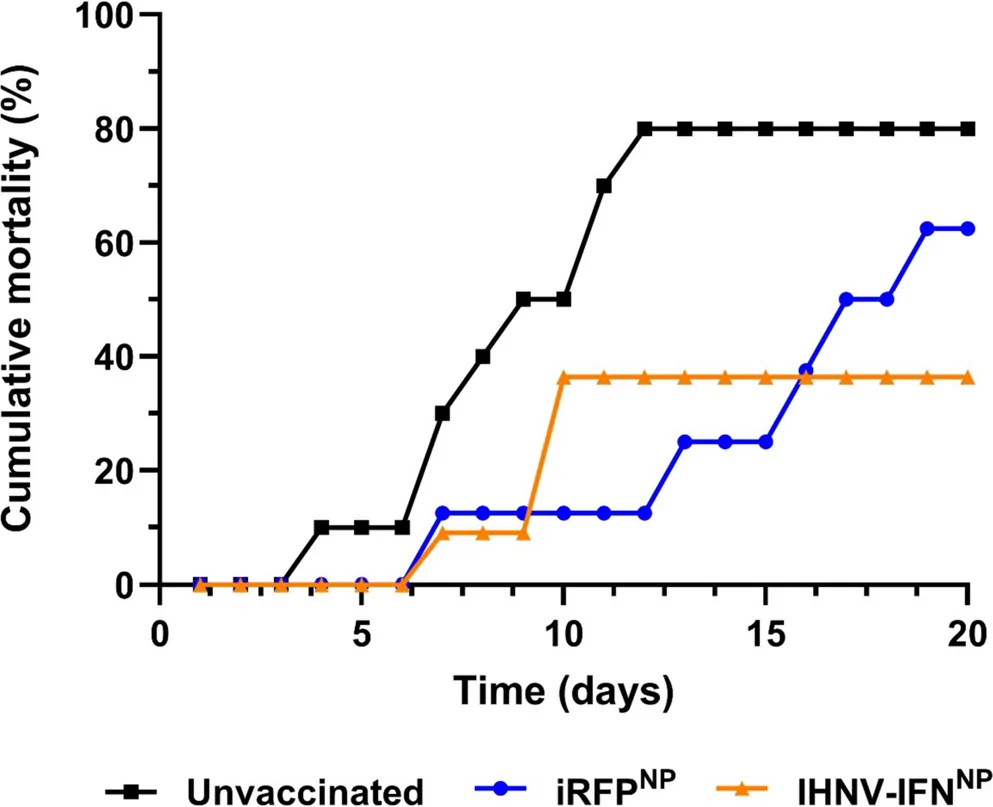
Cumulative mortality rate of rainbow trout after IHNV challenge. Fish were vaccinated by oral intubation with 125 mg/kg of iRFP^NP^ or IHNV-IFN^NP^. Thirty days after vaccination, fish were challenged by intraperitoneal injection with IHNV (1*10^6^ PFU/fish). Number of dead fish in each group was recorded daily

At UHM facilities, a preliminary survival rate assay was performed on IHNV-infected rainbow trout. Unvaccinated fish cumulative mortality raised to 80%, followed by iRFP^NP^ vaccinated fish with a 62.5% and finally by IHNV-IFN^NP^ vaccinated trout with a 36.36% (Fig. 7). Mortality onset occurred at 4 days post-infection (dpi) for the control group, while the other groups delayed it to 7 dpi. After 10 and 12 dpi, IHNV-IFN^NP^ and unvaccinated trout showed no mortality, while iRFP^NP^ kept increasing progressively until the end of the experiment at 20 dpi. Relative Percent Survival (RPS) was 21.9 and 54.5% to iRFP^NP^ and IHNV-IFN^NP^ vaccinated groups, respectively. The same trial was repeated in a second challenge without iRFP^NP^ control finding a cumulative mortality of 20 and 60% in the IHNV-IFN^NP^ and unvaccinated groups, respectively (Supplementary Fig. 3). IHNV^NP^ was not used in these trials due to a low in vitro gene expression induction when compared to the subunit vaccine with the adjuvant module (IFNγ) and comparisons made in between different vaccines previously developed in the group.

## Discussion

Nowadays, there is an urgent need to reduce mortality due biological factors by developing new prophylactic approaches (Sommerset et al. 2024). In this study we present two new recombinant subunit vaccines against Infectious Hematopoietic Necrosis Virus (IHNV). A modular platform against viral disease in fish was used, as in previous developments like viral haemorrhagic septicaemia virus (VHSV) (Aceituno et al. 2025), spring viremia carp virus (SVCV) (Rojas-Peña et al. 2022) and viral nervous necrosis virus (VNNV) (Thwaite et al. 2018, 2020). The platform allows the production of highly stable, modular and cheap subunit vaccines in the form of inclusion bodies (IB) that can be delivered both by injection and oral administration methods.

In the present study, two vaccines have been tested using IHNV G protein and differing only in the addition of an interferon gamma module as an adjuvant. Although this addition, electron microscopy of IHNV^NP^ showed an average length slightly smaller, while average width was similar to IHNV-IFN^NP^ (766.15 ± 123.23 and 610.60 ± 95.22 nm vs. 926.31 ± 185.83 nm and 643.46 ± 80.90 nm, respectively). When compared to previous vaccines, IHNV^NP^ was similar to SVCV^NP^ (693 ± 154 nm and 420 ± 67.5 nm), while IHNV-IFN^NP^ was within the same range of SVCV-IFN^NP^ (982 ± 216.9 nm and 624 ± 104.9 nm) and smaller than VHSV-IFN^NP^ (1205 ± 287 nm and 712 ± 105 nm) (Rojas-Peña et al. 2022; Aceituno et al. 2025). In addition, even using the same production method, protein production yields were greatly protein dependent, as seen in between the two vaccines (206.56 mg/L for IHNV^NP^ and 296.32 mg/L for IHNV-IFN^NP^).

At a functional level, RTS11 tolerated both vaccines, without posing any cytotoxicity at high doses (80 and 160 µg/mL) after 24 h exposure and showing high uptake levels. A similar pattern was recorded by both NPs at dose response and time course assays regarding alive fluorescent cells, measuring only differences in MFI, which cannot be linearly extrapolated to higher particles uptake (Yang et al. 2021). Previous studies assessing VNNV^NP^, IPNV^NP^, VHSV^NP^, SVCV^NP^ and SVCV-IFN^NP^ in ZFL cells and VHSV^NP^ and VHSV-IFN^NP^ in RT-HKM primary culture and RTGutGC cells showed similar MFI and internalization patterns (Thwaite et al. 2018; Rojas-Peña et al. 2022; Aceituno et al. 2025). In addition, Torrealba et al. (2016) assessed in vitro immune response associated to inclusion bodies bacterial-based production methodology using HEK-Blue hTLR4 cell culture. Although inclusion bodies are aggregates of insoluble protein that act as a scaffold incorporating purification-derived residues, like LPS, nucleic acids and bacterial-derived components, immune response promotion could not only be associated to these residues, rather than to a combination of both vaccine components. Furthermore, in the present study, IHNV-IFN^NP^ vaccine has showed a clear pro-inflammatory response promotion compared to the LPS positive control in RTS11 cells gene expression experiments, while iRFP^NP^ and IHNV^NP^ showed a lower expression in *ccl4* and *il1β*.

Meanwhile, internalization patterns imaged by confocal microscopy revealed a colocalization of IHNV-IFN^NP^ and acidic lysosomes. Professional antigen presenting cells (APCs), like macrophages, use exogenous antigen presentation pathway (MHCII) to process antigens that are phagocyted. Thus, lysosome-processed antigen loaded to MHCII trigger pathogen-specific adaptive immunity in the activation of CD4^+^ T cells (Eisenbarth et al. 2021). In addition to that, Nombela et al. (2019) described an alternative activation of endogenous antigen presentation pathway (MHCI) in rainbow trout nucleated RBCs when infected by VHSV, in which antigens were loaded to MHCI molecules through an autophagic pathway (Van Kaer et al. 2019), instead of the typical ubiquitin-proteasome system (UPS). Although MHCI pathway is typically upregulated through IFNγ (Zhou 2009) colocalization and gene expression results presented here, indicated the possibility of a cross-talk between exogenous and endogenous antigen presentation pathways. Hence, gene expression demonstrated a better performance from IHNV-IFN^NP^, showing an upregulation of both innate immune screened genes (*mx*, *vig1*, *ccl4*, *ifngr2*, *ip10*, *il1β*, *mhcI*, and *mhcII*), regardless of *igm*. RTS-11 cells limitation to address adaptive responses is linked to their origin. This cell line mix adherent (macrophage-like) and non-adherent (monocyte-like) cell forms, being both active phagocytes and not showing IgM surface expression (Ganassin and Bols 1998). In addition to that, when searching for professional APCs from fin primary culture, RTS11 was used as a reference culture (Nguyen et al. 2025) and, when comparing cytokine gene expression of different rainbow trout cell lines, such as RTgut, TSS, TPS-2 or RTG-2, among others, RTS11 was clustered with primary leukocytes (Liu et al. 2023). Also, no CD38A transcripts were detected in RTS11, which are associated to B cell differentiation process (Martín et al. 2021). Therefore, RTS11 cell line can be defined as a professional APCs related to innate immunity, rather than a lymphoid cell line related to adaptive response.

Other vaccines with IFNγ module, like SVCV-IFN^NP^ and VHSV-IFN^NP^, also showed an upregulation of antiviral immune response compared to the vaccine without the adjuvant module (SVCV^NP^ and VHSV^NP^) (Rojas-Peña et al. 2022; Aceituno et al. 2025). These results enhance the necessity to add an adjuvant module, like IFNγ, as IHNV^NP^ gene expression did not differ from iRFP^NP^. Moreover, Martin et al. (2007) stimulated RTS11 with recombinant IFNγ and found a strong upregulation of gene expression related to macrophage activation, MHCI antigen presentation pathway, chemokines secretion to attract cytotoxic T cell and Th1 adaptive response. When comparing recombinant IFNγ *versus* a transfected IFNγ plasmid, the recombinant one promoted a higher inflammatory and humoral immunity, while activated a lower cellular immunity (Wang et al. 2021). In our case, the IFNγ module was linked to the antigenic protein and was produced in the form of inclusion bodies, thus cytokine addition as vaccine adjuvant has been described to be cytokine type and dose dependant (Cao et al. 2017). Other studies have used chemokine CK6 coupled to truncated IHNV-G protein expression in *Lactobacillus casei* (Sun et al. 2022), demonstrating an expression increase of antiviral-related cytokines that helped resist viral infection. While Gorgoglione et al. (2023) used a recombinant subunit vaccine of IHNV-G protein together with complement C5a as adjuvant and chemoattractant of leukocytes, showing a low level of antiviral protection compared to glycoprotein alone. Moreover, higher doses of cytokines can promote tolerogenic responses (Volpi et al. 2013).

Currently, there is only one DNA vaccine commercially available against IHNV (APEX-IHN, Merck Animal Health). Also, the first mRNA vaccine has been tested in rainbow trout with an RPS of 84.61% (Wang et al. 2025). Nonetheless, most of the studies in IHNV vaccine development focus in DNA vaccines (Alonso et al. 2011; Xu et al. 2017; Jones et al. 2020; Marsella et al. 2022; Wu et al. 2023; Zhao et al. 2024; Doumayrou et al. 2025), usually based in intramuscular injection, which is labour intensive and not quite suitable for fries at industrial scale. On the other side, few studies have been done using subunit vaccines. Engelking and Leong (1989) were the first in vaccinate rainbow trout and sockeye salmon by immersion with a purified G protein subunit vaccine produced in bacteria. More recently, Verjan et al. (2008) injected intraperitoneally (ip) a nonglycosylated recombinant subunit vaccine from solubilized inclusion bodies producing an increase in innate immune response mainly mediated by IFNγ. Using the same administration route, Hua et al. (2020) vaccinated rainbow trout with a truncated IHNV glycoprotein, producing a humoral immune response with a peak at 28 dpi and achieving a 60% survival rate after a 14-day infection challenge. Lastly, Luo et al. (2026) obtained similar survival results (63%) with a recombinant IHNV-G protein vaccine, although the most interesting results came from the synergistic effect of microbiota regulation by vaccine administration. In our case, vaccine design aimed to orally administered recombinant subunit vaccines due to its low labour, low cost, less stressful and more convenient industry scale approach. Nonetheless, oral vaccines face other challenges, such as gastrointestinal pH barrier, dose determination and hindgut absorption to trigger a humoral and cellular immune responses. To our knowledge, several attempts had been made to produce orally administered vaccines against IHNV, some based in DNA plasmid encapsulated in different delivery vectors, such as PLGA (Poly(D, L-Lactic-Co-Glycolic acid) (Adomako et al. 2012), alginate microspheres (Ballesteros et al. 2015), chitosan or Montanide™ GR 01 (Shao et al. 2026), developing lower protection in orally administered than in intramuscular injection. Other approaches were done using bacterial (Sun et al. 2022) or fungal (Zhao et al. 2017) as a vector to colonize rainbow trout intestine microbiota and produce IHNV-G protein in the gut. Results from these studies showed a cumulative mortality in bacteria around 40% and 52% in yeast after infection challenge. In our case, survival rate assay of IHNV-IFN^NP^ orally intubated trouts showed a RPS of 54.5% after 20 dpi and 75.3% after 32 dpi (Fig. 7 and Sup. Figure 3, respectively). Although RPS values, when assessing adaptive immunity promotion, only partial promotion was found. Gut gene expression showed an upregulation of *igm* and a tendency to increase expression in *igt* (Fig. 6A) as has been reported in other studies (Ballesteros et al. 2013; Rombout et al. 2014). Most of mucosal adaptive immunity genes evaluated did not show significant differences, but a tendency in *mhcII* and *il10* (Sup Fig. 1). Similarly, specific anti-IHNV IgM titer tended to increase, but not significantly (Fig. 6B and Supplementary File 2). As commented above, IHNV^NP^ subunit vaccine was not incorporated to the trial due to low antiviral response in RTS11 gene expression assays. Furthermore, we associate iRFP^NP^ mortality pattern to non-specific immune stimulation delaying the mortality onset, which is similar to SVCV infection challenge presented by Rojas-Peña et al. (2022). Other trials, like SVCV^NP^ and VHSV^NP^, have demonstrated that IFNγ adjuvant module enhances antiviral and immune response, both in vitro and in vivo and both in oral intubated and in-feed administered (Rojas-Peña et al. 2022; Aceituno et al. 2025). These results demonstrate the viability of orally prime vaccinating with relatively high doses. Nonetheless, a more detailed study of dose-dependent response should be developed to determine the sweet spot for this route of vaccination using inclusion bodies. Thus, a recent study has shown that primary vaccination route is crucial to following booster doses to keep fish protection high (Abouismail et al. 2026). The same study brought attention to an age-dependent component of mucosal vaccination. As nasal mucosal barrier thickness increases with age, it reduces vaccine immunization effectivity. According to the literature (Tatner and Manning 1983), rainbow trout develops its immunity at around 14 days post-hatching. Other studies (Salinas et al. 2015; Marana et al. 2020) had assessed rainbow trout early immunization in individuals ranging from 1.5 to 5 g and through different mucosal immunization methods, like immersion, bath, nasal and/or oral. In this sense, we have demonstrated that oral vaccination of young rainbow trout with body weight ranging from 3 to 3.5 g is viable with partial protection against IHNV. Nonetheless, it could be interesting to assess age-dependent component of mucosal vaccination, the effect of oral vaccine administration as primary vaccination method and its effect on subsequent booster doses and/or when applying other boosting methods.

In summary, two recombinant subunit vaccines against IHNV were evaluated in the present stud, being different only by the addition of an IFNγ adjuvant module in one of them. At high doses, both vaccines were successfully up taken by RTS11 cells. However, adjuvant module enhanced the expression of an in vitro antiviral response in both innate immune response genes and was colocalized in acidic lysosomes. After oral intubation, partial vaccine protection was achieved with a RPS of 54.5 and 75.3%, as well as potentially inducing systemic and mucosal immune responses, as suggested by the tendency of upregulation of *igt* gene associated with intestinal mucosal immunity following vaccination, demonstrating the suitability of the vaccine and the vaccine administration method. Further studies are needed to determine whether IHNV-IFN^NP^ can protect rainbow trout fingerlings against IHNV when in-feed orally administered, its use as prime and booster vaccination and the best dose for oral vaccination at different development stages.

## Supplementary Information

Below is the link to the electronic supplementary material.

Supplementary Material 1 (DOCX 16.8 KB)

Supplementary Material 2 (DOCX 131 KB)

Supplementary Material 3 (DOCX 114 KB)

Supplementary Material 4 (DOCX 137 KB)

Supplementary Material 5 (DOCX 219 KB)

## Data Availability

Experimental data will be available upon request.
