# Supplementary material for: Development of an Oral Subunit Vaccine to Protect Rainbow Trout Against Infectious Hematopoietic Necrosis Virus (IHNV)
Source: Mar Biotechnol (NY). 2026 Nov 12;28(6):183. doi: 10.1007/s10126-026-10728-w (PMC13759613; doi:10.1007/s10126-026-10728-w)
Supplement: Supplementary file 1 [file 10126_2026_10728_MOESM1_ESM.docx]

| **Gene** | **Sequence (5´-3´)** | **Accession** | **Reference** |
| --- | --- | --- | --- |
| ***ef1-α*** | FW_ CAAGGATATCCGTCGTGGCA | NM_001124339.1 | Tachi et al. (2010) |
|  | RV_ ACAGCGAAACGACCAAGAGG |  |  |
| ***b-actin*** | FW_ CAAGGATATCCGTCGTGGCA | XM_014194536.2V | Valenzuela et al., (2023) |
|  | RV_ ACTCCTGCTTGCTGATCCAC |  |  |
| ***mx*** | FW_ ATGCCACCCTACAGGAGATGAT | NM_001171901.1 | Thwaite et al. (2018) |
|  | RV_ TGCAGCTGGGAAGCAAACTCC |  |  |
| ***vig1*** | FW_ AACGCTGGGGAGAACAGTCT | NM_001124253.1 | Thwaite et al. (2018) |
|  | RV_ TCCCCTCTCGGCAATCCA |  |  |
| ***ccl4*** | FW_TGTTCACCCCTCGTCTTGCT | NM_001124489.2 | Thwaite et al. (2018) |
|  | RV_ ACATTTCTTCGGTCCGCTTG |  |  |
| ***ifngr2*** | FW_ GAGTCCATCCAAGAGTCAGAAAG | EU244877.1 | Xu et al. (2016) |
|  | RV_ CCCACTACCAAGACACCCAAC |  |  |
| ***ip10*** | FW_ AGCCACTCCTGCAGGAACAT | AJ417078.1 | Zou et al. (2005) |
|  | RV_CACTTCTTCCCTTCTCCGTTCC |  |  |
| ***il-1β*** | FW_ GGAGGCAGCAGCAGCTACCACAAA | XM_036979104.1 | Clark et al. (2021) |
|  | RV_ CCGATTTGGAGCAGGACAG |  |  |
| ***mhcI*** | FW_ GACAGTCCGTCCCTCAGTGT | AF287485.1 | Chaves-Pozo et al. (2010) |
|  | RV_ CTGGAAGGTTCCATCATCGT |  |  |
| ***mhcII*** | FW_ TGCCATGCTGATGTGCAG | AF115533.1 | Jørgensen et al. (2009) |
|  | RV_ GTCCCTCAGCCAGGTCACT |  |  |
| ***igm*** | FW_ CTTGGCTTGTTGACGATGAG | S63348.1 | Andersson et al. (1993) |
|  | RV_ GGCTAGTGGTGTTGAATTGG |  |  |
| ***igt*** | FW_CATCAGCTTCACCAAAGGAAGTG | AY870268.1 | Attaya et al. (2020) |
|  | RV_TCACTTGTCTTCACATGAGTTACCC |  |  |
| ***tgfβ*** | FW_GCTGCCGACAACAAAGATAATG | NM_001281368.2 | Maehr et al. (2012) |
|  | RV_GTGCCAGCGATCTCCATGT |  |  |
| ***cd4*** | FW_ GTGTGGAGGTGCTACAGGTTTTTTC | AY973028 | Attaya et al. (2020) |
|  | RV_ATCGTCACCCGCTGTCTGTG |  |  |
| ***cd8*** | FW_CAAGTCGTGCAAAGTGGGAAA | AF178053 | Attaya et al. (2020) |
|  | RV_TCTGTTGTTGGCTATAGGATGT |  |  |
| ***foxp3*** | FW_CCCAGAACCGAGGTGGAGTGT | FM883710 | Attaya et al. (2020) |
|  | RV_TGACGGACAGCGTTCTTCCA |  |  |

Table S1 Rainbow trout primers used for RT-qPCR
