# Supplementary material for: Development of an Oral Subunit Vaccine to Protect Rainbow Trout Against Infectious Hematopoietic Necrosis Virus (IHNV)
Source: Mar Biotechnol (NY). 2026 Nov 12;28(6):183. doi: 10.1007/s10126-026-10728-w (PMC13759613; doi:10.1007/s10126-026-10728-w)
Supplement: Supplementary file 2 [file 10126_2026_10728_MOESM2_ESM.docx]

Fig. S1 Gene expression of gut after oral intubation with PBS 1x (Control, black), iRFP^NP^ (blue) and IHNV-IFN^NP^ (orange). Data are mean ± SD (n=10). Differences between means were analysed by a one-way ANOVA with Dunnett’s multiple comparisons test, treatments versus control, unpaired one-sided t-test with Welch’s correction for unequal variances or unpair nonparametric Mann-Whitney test. Significance levels **p < 0.05; **p< 0.01; ***p < 0.001; ****p < 0.0001*
