# Supplementary material for: Development of an Oral Subunit Vaccine to Protect Rainbow Trout Against Infectious Hematopoietic Necrosis Virus (IHNV)
Source: Mar Biotechnol (NY). 2026 Nov 12;28(6):183. doi: 10.1007/s10126-026-10728-w (PMC13759613; doi:10.1007/s10126-026-10728-w)
Supplement: Supplementary file 3 [file 10126_2026_10728_MOESM3_ESM.docx]

Fig. S2 Cumulative mortality rate of rainbow trout after IHNV challenge. Fish were vaccinated by oral intubation with 125 mg/kg of IHNV-IFN^NP^. Thirty days after vaccination, fish were challenged by intraperitoneal injection with IHNV (1*10^6^ PFU/fish). Number of dead fish in each group was recorded daily
