# Supplementary material for: Development of an Oral Subunit Vaccine to Protect Rainbow Trout Against Infectious Hematopoietic Necrosis Virus (IHNV)
Source: Mar Biotechnol (NY). 2026 Nov 12;28(6):183. doi: 10.1007/s10126-026-10728-w (PMC13759613; doi:10.1007/s10126-026-10728-w)
Supplement: Supplementary file 4 [file 10126_2026_10728_MOESM4_ESM.docx]

Fig. S2 Presence of specific anti-IHNV IgM sera of rainbow trout. Fish were intubated on 3 consecutive days with a cumulative dose of 125 mg/Kg of NPs. Sera was obtained 30 days post-immunization from IHNV-IFN^NP^ vaccinated (orange), iRFP^NP^ (blue) and IHNV infection survival fish positive control (red). Data are mean ± SD (n=3).
