# Supplementary material for: Development of an Oral Subunit Vaccine to Protect Rainbow Trout Against Infectious Hematopoietic Necrosis Virus (IHNV)
Source: Mar Biotechnol (NY). 2026 Nov 12;28(6):183. doi: 10.1007/s10126-026-10728-w (PMC13759613; doi:10.1007/s10126-026-10728-w)
Supplement: Supplementary file 5 [file 10126_2026_10728_MOESM5_ESM.docx]

Fig. S4 RTS11 cells flow cytometry gating strategy. Alive cells (blue), Dead cells (red) and fluorescent-labelled NPs uptaken by alive cells (green). In the X-Axis Forward Scatter (FSC) and in the Y-Axis Side Scatter (SCC).
